# The recovery of parabolic avalanches in spatially subsampled neuronal networks at criticality

**DOI:** 10.1101/2024.02.26.582056

**Published:** 2024-02-28

**Authors:** Keshav Srinivasan, Tiago L. Ribeiro, Patrick Kells, Dietmar Plenz

**Affiliations:** Section on Critical Brain Dynamics, National Institute of Mental Health, Bethesda, MD 20892, USA

**Keywords:** Neuronal avalanches, E/I balanced neural network, subsampling, thresholding, scaling exponent, criticality, ongoing activity, mouse, frontal cortex, 2-photon imaging

## Abstract

Scaling relationships characterize complex systems at criticality. In the brain, these relationships are evident in scale-invariant activity cascades, so-called neuronal avalanches, quantified by power laws in avalanche size and duration. At the cellular level, neuronal avalanches are identified in spatially distributed groups of neurons that participate in cascades of coincident action potential firing. Such spatiotemporal synchronization is central to theories on brain function, yet scaling relationships in avalanche synchronization have been challenging to study when only a fraction of neurons is observed, underestimating avalanche properties. Here, we study these biases from fractional sampling in an all-to-all, balanced network of excitatory and inhibitory neurons with critical branching process dynamics. We focus on the growth of mean avalanche size with avalanche duration. For parabolic avalanches, this growth is quadratic, quantified by the scaling exponent, χ=2, which signifies rapid spatial expansion of coincident firing within a relatively short period of time. In contrast, χ<<2 for fractionally sampled networks. We show that temporal coarse-graining combined with a threshold for the minimally required coincident firing in the network recovers χ=2, even when sampling as few as 0.1% of the neurons. In contrast, a commonly proposed ‘crackling noise’ approach fails to recover χ under those conditions. Our approach robustly identifies χ=2 for ongoing neuronal activity in frontal cortex of awake mice using cellular 2-photon imaging. Our findings demonstrate how to correct scaling bias from fractional sampling and identifies rapid, scale-invariant synchronization of cell assemblies in the brain.

## AUTHOR SUMMARY

In the brain, groups of neurons often fire together which is considered essential for normal brain function. A particular form of coincident firing has been identified for “neuronal avalanches“ which are scale-invariant cascades of neuronal activity that rapidly engage large numbers of neurons. However, studying these avalanches when observing only a few neurons in the network is challenging. We used simulations and recordings from ongoing brain activity to explore how to overcome this challenge. We found that adjusting our temporal resolution and focusing on increasingly larger avalanches allows us to accurately detect rapid, parabolic expansions of these patterns even when examining a minuscule fraction of neurons. This identifies a general rule for how the brain operates best in engaging neurons to fire together, despite the limitations in our observations.

## Introduction

Complex systems, composed of many local components or agents that interact weakly, often exhibit event cascades that cover a wide range of scales in both space and time. Such scale-invariant cascades, typically identified by power-law distributions in their duration and size – defined as the total activity observed during its lifetime, have been observed in many real systems, including solar flares [1], earthquakes [2, 3], superconductors [4], sandpiles [5], forest fires [6], and in the brain in the form of neuronal avalanches [7–9]. The scaling of such system cascades, specifically how their mean size grows with their duration, has been particularly informative as to the potential underlying dynamics of cascade generation. For example, if local system components activate independently at a constant rate, the mean size, <S>, of observed cascades will scale linearly with duration, T, exhibiting a scaling exponent of χ≅1, i. e., temporally contiguous system events reflect a constant, yet random probability of their continuation. Values of χ≅1 are often found near a 1^st^ order phase transition [10–12]. Conversely, if cascades exhibit χ=2, their mean growth is rapid and parabolic as can be found at the second-order phase transition of a critical branching process, supporting local system interactions [13]. In general, values of χ>>1 are of particular interest as they suggest the ability of a system to bind spatially distributed events rapidly and selectively into diverse internal system configurations which is generally considered beneficial for a complex adaptive system such as the brain (e. g., [14, 15]).

Here, we focus on neuronal avalanches, which were first shown to spontaneously emerge in isolated brain networks, i. e. acute cortex slices and long-term cortex cultures [7]. By measuring locally synchronized neuronal activity using the local field potential (LFP) and tracking the spatiotemporal spread of LFP cascades with microelectrode arrays, power-law distributions for both size, S, and duration, $T,\;P(S)\sim S^{-\alpha }$ and $P(T)\sim T^{-\beta }$ were revealed, with exponents α≈3/2 and β≈2 respectively, in addition to a critical branching parameter of σ≅1, and significant correlation of neuronal activity within and between [16, 17]neuronal cascades (for review see [18]). These hallmarks of fast, neuronal synchronization in the form of avalanches were subsequently confirmed in the normal brain for ongoing and evoked LFP cascades in the cortex of nonhuman primates [19–22] and in humans using magnetoencephalography [23, 24]. Their sensitivity to even slight pharmacological alterations in the balance of excitation and inhibition [7, 18, 22, 25] and changes during pathological conditions such as epilepsy [26–29] or changes in brain states such as sleep [30, 31] and sleep deprivation [32–35], firmly indicate that these avalanches represent neuronal synchronization of cell assemblies in the critically tuned, normal brain.

To understand avalanche synchronization mechanistically, the contribution of single neuron firing to avalanches needs to be properly identified. This task is extremely challenging as studies in nonhuman primates and isolated slice preparations show that neurons participate sparsely and highly selectively in even spatially extended LFP avalanches [22]. Accurate reconstruction of avalanche synchronization from single neuron activity, therefore, requires the appropriate sampling of a significant portion of the brain network which poses an enormous technical challenge in neuroscience. Conversely, spatial subsampling, that is, sampling of only a fraction of neurons, is limited in identifying neuronal synchronization largely by failing to identify avalanche continuation in non-sampled neurons. This underestimates avalanche duration and size, biasing outcomes towards expectations for decorrelated, random processes [9, 36, 37]. Accordingly, simulations of avalanche generating critical branching processes and fractional sampling of neurons reduces the expected growth in mean avalanche size with avalanche duration, χ, from χ=2, to a value of χ closer to 1 – 1.3 [38–40]. Not surprisingly, estimates of χ≅1.3 were reported for fractionally sampled neuronal spikes of potential avalanche activity *in vivo* [39, 41–43] and *in vitro* [44].

If the exact start and end times of avalanches are known, for example in simulations under the assumption of ‘separation of time scales’, spatially subsampled avalanches can be easily corrected for by temporal integration thereby recovering the correct critical exponents in size and duration [40] (for a review, see also [45]). Avalanche times, however, are largely unattainable in experimental data for which only a small fraction of neurons is observed and temporal integration eventually will degenerate avalanche scaling by indiscriminately concatenating successive avalanches [9]. Recently, Capek & Ribeiro et al. [9] demonstrated that, even when the exact times of avalanches are not known, χ=2 can be recovered for subsampled excitation-inhibition (E-I) balanced networks of integrate-and-fire neurons which are known to belong to the mean-field directed percolation (MF-DP) universality class [46, 47]. This rescue requires (1) reducing uncorrelated neuronal action potential firing and (2) using temporal coarse-graining with an increase in minimal synchronization requirements to identify synchronized population activity in the network. Under these conditions, the authors could show χ=2 *in vivo* 2-photon imaging recordings of cellular activity with high signal-to-noise ratio for sensory evoked and spontaneous spike avalanches in frontal and sensory cortices of awake mice in line with their simulations [9]. This is in line with a recent EEG study in humans that demonstrated χ=2 [48].

In the current work, we extend our previous investigations of rescuing χ in subsampled, critical networks. We systematically study the rescue of the scaling exponent χ to its critical value of 2 in a continuously driven, subsampled all-to-all DP-model [46] for which we demonstrate the equivalence of fractional sampling and thresholding. This allows us to identify a robust domain within the ‘temporal coarse graining’ vs. thresholding parameter regime over 4 orders of magnitude of fractional sampling. We identify this domain in cellular 2-photon imaging data of the frontal cortex of awake mice. We further show that the ‘crackling noise’ relationship and related measures which can predict χ [49–51] fail under conditions of subsampling. Our findings demonstrate the pitfalls of assessing avalanche synchronization when only parts of a critical network are observed and demonstrate the regime of potential scaling rescue for synchronization in the form of parabolic avalanches under experimental conditions of fractional sampling.

## Results

**Figure 1.**
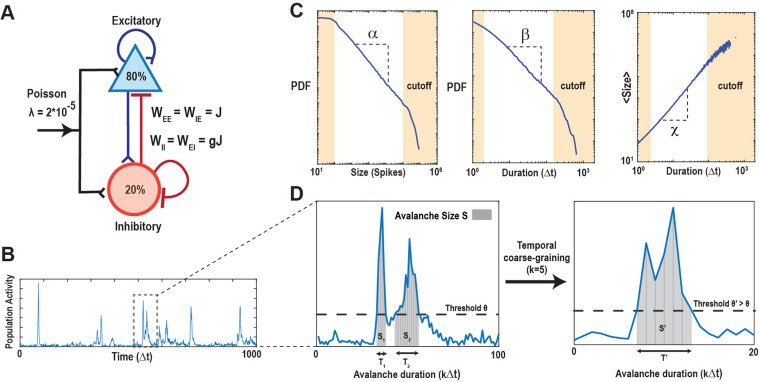
Schematics of the balanced E/I-model and identification of suprathreshold sequences in population spiking activity with respect to minimal coincident spiking threshold and temporal coarse graining. (**A**) Schematic of the neuronal network consisting of N = 10^6^ neurons (80% excitatory and 20% inhibitory) with a low external Poisson drive of rate λ = 2*10^−5^ per time step per neuron. The E/I balance is controlled by g, which scales the inhibitory weight matrices (W_II_, W_EI_) as a function of the excitatory weight matrices (W_EE_, W_IE_). If not otherwise stated, we set g=3.5 to obtain critical dynamics (see Methods). (**B**) High variability in intermittent population activity characterizes critical dynamics. Snapshot of the summed neuronal spiking activity as a function of time. (**C**) Duration, size and scaling of avalanches in the critical model follow power-laws with corresponding exponent estimates α,β and χ. Note that the external Poisson drive and the finite size of the network introduce lower and higher cut-offs, respectively (*beige areas*). (**D**) Zoomed population activity from *B*. At the original temporal resolution Δt and given the coincident spiking threshold, θ (*left*), we can identify two sequences of suprathreshold activity, $S_{1}$ and $S_{2}$ with durations $T_{1}$ and $T_{2}$, respectively. Temporally coarse-graining the population activity (right; k=5, binning the data into new bins of 5 time points) and increasing the threshold to θ′>θ, absorbs $S_{1}$ and $S_{2}$ into a new suprathreshold activity period $S^{\prime }$ with duration T′.

Neuronal avalanches were studied using a critically balanced Excitatory-Inhibitory (E-I) model with all-to-all connectivity consisting of one million neurons driven externally using independent, stationary Poisson processes [46] (Fig. 1A). The external driving triggered an average of 20 neurons to fire per time step, Δt, which intermittently induced spreading of neuronal activity in the network. Simulations spanned across 10^8^ time steps. An avalanche was defined as a series of contiguous time periods, T, of aggregated neuronal activity surpassing a predetermined threshold, θ, over a specific number of time steps, k (Fig. 1B – D). Our analytical approach was characterized by two basic parameters: k⋅Δt and θ, which were explored in the context of the subsampling fraction of observed neurons, f, which ranged from 1 out of every 10,000 neurons observed (f=0.01%) to all neurons observed (f=100%). We focused in our analysis on continuous sequences of coincident neuronal firing above θ, i. e., suprathreshold activity (Fig. 1,B D). We identified the statistical distribution of sequence size, S, and duration, T, as a function of θ,f, and k and estimated the corresponding size exponent, α, and duration exponent, β, within the constraints of the lower and upper cut-offs (Fig. 1C; see Methods; [52, 53]). In our critical model, these distributions take on the form of power laws at k=1,f=100% and θ=100, the defining characteristics of avalanches [7]. The scaling exponent, χ, governs the growth in average avalanche size, 〈S〉, for a given duration, T,

(1) $$\langle S\rangle \sim T^{\chi },$$

and accordingly, we estimated the scaling factor χ from the slope of the average avalanche size, 〈S〉, for a given avalanche duration, T, outside the minimal and maximal cut-off in logarithmic scales (Fig. 1C, see Methods). In critical systems, it has also been found that χ relates to the critical exponents $\alpha^{\prime }$ and $\beta^{\prime }$ as

(2) $$\chi_{cn}=\frac{\left(\beta^{\prime }-1\right)}{\left(\alpha^{\prime }-1\right)}$$

(‘cn = crackling noise’ [49–51]). For comparison, we therefore calculated both $\chi_{cn}$ and

(3) $$DCC=\chi -\chi_{cn}$$

from our slope estimates α and β. This latter construct has been suggested as a distance measure from a critical point implying that the network dynamics is close to criticality with vanishing DCC (e.g. [54]).

### High coincident firing thresholds underestimate χ in the fully sampled model

**Figure 2.**
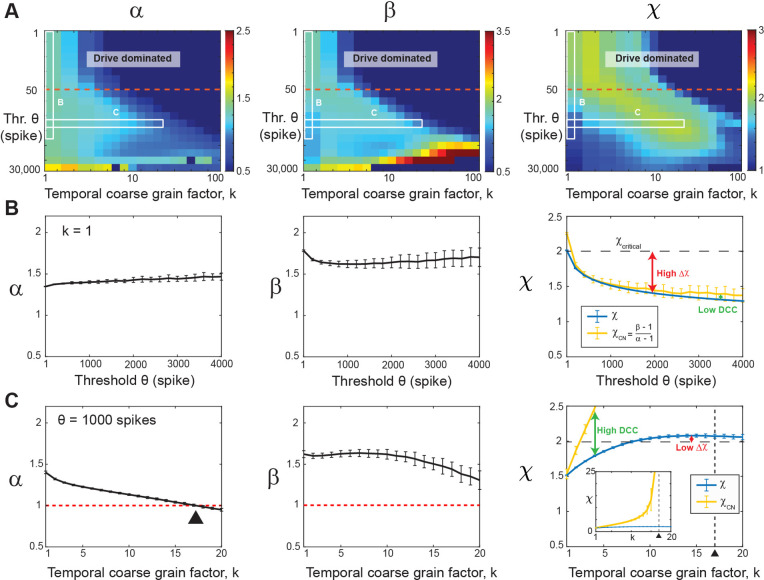
Increasing the threshold in the fully sampled model underestimates the scaling exponent, χ which can be rescued by temporal coarse graining. (**A**) Consolidated view of the avalanche size (α), duration (β), and scaling (χ) exponents as a function of threshold (θ) and temporal coarse-graining factor, k(f=100%, fully sampled model). *Drive dominated*: Low threshold regime (*above the red broken line*) dominated by external Poisson drive. *White frames*: Parameter regions displayed in *B* and *C*. (**B**) At the highest temporal resolution (k=1), high thresholds underestimate the scaling exponent, χ, as well as $\chi_{CN}$, with DCC remaining low. Size exponent, α (*left*), duration exponent, β (*middle*), scaling exponent, χ, and expected crackling noise relation, $\chi_{CN}$, (*right*) as a function of θ. (**C**) At high threshold (θ=1000), temporal coarse graining recovers χ, but the $\chi_{CN}$ exhibits a singularity leading to high DCC. Size exponent, α (*left*), duration exponent, β (*middle*), as a function of coarse graining factor, k, (θ=1000). α passes through 1 (*red broken line; black triangle*), which causes a singularity in $\chi_{CN}$. (*right*). Note that the scaling exponent, χ, stabilizes to a value of 2 (*right, black dashed line*), whereas $\chi_{CN}$ grows until it passes through a singularity at the temporal coarse graining value of k=17 (*vertical broken line; triangle*; see also inset).

In systems characterized by a clear separation of timescales and an absence of noise, the necessity for a coincident firing threshold diminishes. However, within real systems lacking such temporal separation and affected by significant intrinsic and measurement noise, the statistical analysis becomes intricately tied to our selection of threshold values. Setting the threshold too low poses the risk of incorporating substantial noise from the system, while setting it too high leads to data sparsity, resulting in the exclusion of a significant portion of the system’s activity. To emulate the noisy environment and absence of timescales separation between avalanche generation and propagation inherent in real systems, we introduce a minimal external Poisson drive in our simulations. This Poisson drive establishes a noise floor within the spiking activity, serving as the origin of avalanches within the system. This is clearly visible in the size distribution of avalanches, which exhibits a power law only for avalanche sizes of $S_{0}>50-100$ spikes up to the finite-size of the network (cf. Fig. 1C, *middle*) [46]. To isolate avalanches above the noise floor (i. e., $S>S_{0}$), we first studied the dependence of α,β,χ on θ and k in the fully sampled network (f=100%; Fig. 2). In Fig. 2A, we show an overview in the form of color maps for all three parameters when increasing θ. At the highest temporal resolution (k=1), we observed that α and β, increase with θ>100, with a corresponding rapid decrease of χ below its expected value of 2 (cf. Fig. 2B). We conclude that high thresholds in weakly driven, noisy networks bias towards longer suprathreshold periods, significantly underestimating the scaling exponent for the critical system.

A similar underestimation holds true for the $\chi_{cn}$ value. Inserting the corresponding values of exponents extracted from our fully sampled system for small thresholds, we obtain $\chi_{cn}=2$, in line with expectations for the scaling exponent for the mean-field directed percolation (MF-DP) universality class [55]. However, $\chi_{cn}$ drops significantly below 2 when θ increases, largely following the dependence of β on θ (*c.f.* Fig. 2A). We also observe a small systematic underestimation of $\chi_{cn}$ with respect to χ (Fig. 2B), which we attribute largely to the error-bounds of our fitting procedure. We conclude that avalanche scaling will be underestimated even in fully sampled networks if there are technical constraints on minimal coincident spike counts, which require the use of high thresholds.

### Temporal coarse-graining rescues χ at high thresholds in the fully sampled model

Next, we show that temporal coarse graining rescues χ in the presence of a high threshold in fully sampled networks. This is shown in Fig. 2C, in which χ approaches 2 for k>8 at θ=1000 (see also corresponding projections in Fig. 2A). In contrast, the size and duration distributions exhibit a gradual reduction in steepness when k increases, due to concatenating subsequent cascades, in line with experimental findings [7, 19, 40, 56]. In the model, this eventually causes α to cross the value of 1 (Fig. 2C), which forces $\chi_{cn}$ to undergo a singularity (Equation 2; Fig. 2C, *inset*). Accordingly, we find a rapid breakdown in the agreement between χ and $\chi_{cn}$ with increase in k, with diverging *DCC* (Fig. 2C). We conclude that while temporal coarse-graining can mitigate the bias introduced by thresholding in the fully sampled model, the *DCC* is an unreliable estimate for avalanche scaling even in fully sampled networks under high thresholding conditions.

### Scaling collapse demonstrates equivalence of thresholding and subsampling in the critically balanced all-to-all E/I-model

**Figure 3.**
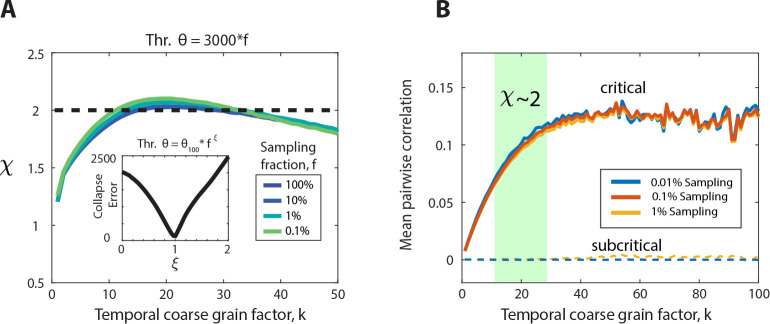
Equivalence in threshold and fractional sampling and increase in mean pair-wise correlation among neurons in the critical model with temporal coarse graining. (**A**) Collapsed scaling exponent curves for different values of the sampling fraction, f, ranging from 100% to 0.1% for a threshold that is scaled by the sampling fraction as θ=3,000*f (i.e., a threshold of 3,000 spikes for the fully sampled model translates to a threshold of 3 spikes for the 0.1% sampled model). *Inset*: The total collapse error as a function of collapse exponent, ξ. The error is taken over multiple different curves with θ ranging from 100 to 100,000. The error shows a clear minimum at ξ=1, indicating the threshold can be scaled proportionally with the sampling fraction to obtain the best collapse. (**B**) The rescue of χ=2 correlates with an increase of the delayed mean pair-wise correlation among neurons, which is independent of the fraction of neurons sampled and not found in subcritical networks. The temporal coarse graining regime of k for which χ=2 (*green area*) is not predicted by the pair-wise correlation among neurons.

Both, the increase in coincident spiking threshold θ as well as the decrease in sampling fraction f underestimate the scaling exponent χ due to prematurely terminating avalanches by unobserved network activity. On the other hand, temporal coarse graining allows for concatenating sequential activity periods potentially recovering the proper duration for a subset of avalanches. We therefore examined the impact of temporal coarse graining on thresholding and fractional sampling. To achieve this, we first collapsed the χ vs. k curves over a wide range of θ (10^2^ – 10^5^) by scaling θ with an exponent of f,ξ (see Methods). We found that the corresponding functions, obtained for different values of f, exhibited a minimum total collapse error for ξ=1, demonstrating a linear relationship between θ and f in our all-to-all model. This implies that a fully sampled network (10^6^ neurons) with θ=3,000 spikes is qualitatively equivalent to a 0.1% sampled model (1,000 neurons) with θ=3 spikes (Fig. 3A). This equivalence between θ and f allows us to rescale the recovery of χ for different f in the corresponding (k,θ)-plane for ease of comparison (see below).

### The rescue of χ=2 correlates with a recovery of mean pair-wise correlations

Subsampling as well as decorrelation of spiking activity have been shown to reduce the expected growth in mean avalanche size with avalanche duration from χ=2, to a value of χ closer to 1 – 1.3 [38–40]. We therefore studied how the delayed mean pair-wise correlation, $<C_{ij}(1,k)>$ changes with temporal coarse graining when recovering χ (see Methods). Temporal coarse graining increases $<C_{ij}(1,k)>$ in the critical balanced E/I-network and saturates at high k, independent of the amount of fractional sampling (Fig. 3B). The increase in correlation did not predict the temporal coarse graining value of k at which χ=2 and was not found in subcritical networks, for which the average pairwise correlation remains near zero and independent of k (Fig. 3B; g=3.75). These findings demonstrate that χ=2 identifies the expansion of coincidental firing among groups of neurons, which is not captured by a particular value of $<C_{ij}(1,k)>$, a measure limited to studying pairs of neurons.

### Subsampling and spike-resolution introduce unobservable regions of scaling exponents

**Figure 4.**
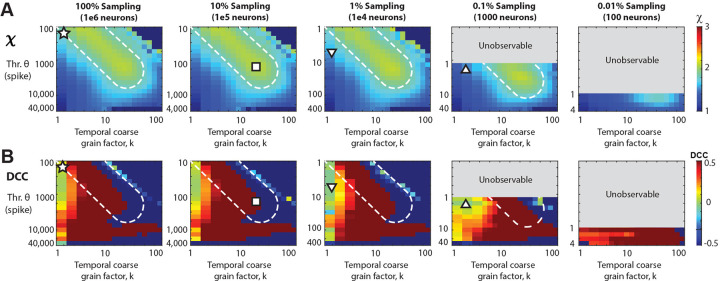
Temporal coarse -graining identifies a robust rescue regime for χ=2 covering 0.1% - 100% fractional sampling at decreasing thresholding level, which is absent for the *DCC* (**A**) Consolidated view of χ and DCC as a function of θ and k for different values of f. For f=100%, χ≅2 for low θ and k (*star*) but as we make the data sparser by increasing the threshold (or reducing the sampling), we need a higher coarse graining factor, k, to compensate and rescue χ back to 2 (*square*). *White dotted region*: visual guide for χ close to 2 for increasing θ and k. The grey parts of the plots for f=0.1% and 0.01% respectively are unobservable parameter regions since they would require fractional thresholds, below the 1 spike minimum resolution of the model. At f=0.1% sampling, χ can be rescued to a value of 2, but not for more severe subsampling, f=0.01%. For triangles see *B*. (**B**) The *DCC* remains close to 0 only for low k, and quickly breaks down at higher k values. For the fully sampled model, there exists a region with the correct scaling exponent χ≅2 as well as low *DCC* (*star* in *A* and *B*) However, as we move to lower sampling, this region of correspondence becomes more and more difficult to maintain. For f=1% and 0.1%, regions at low k, and moderate threshold (or equivalently lower sampling) remain a low *DCC*, but underestimate the true scaling exponent, χ (*up* and *down triangles* respectively).

By taking advantage of the trade-off between thresholding and sub-sampling (see Subsection above), we now can rescale our k vs. θ parameter space by f⋅θ to account for different fractional sampling. In the consolidated analysis (Fig. 4), we demonstrate reasonable agreement with the rescue of χ=2 from 0.1% - 100% fractional sampling. When we increase the threshold (or equivalently, decrease f), we observe a deviation from χ=2, yet the exponent can be reliably restored at higher values of k (Fig. 4, white broken lines). On the other hand, reducing f eventually introduces unobservable (k,θ)-regions which arise from the 1 spike resolution of the model excluding fractional thresholds. We note that even when sampling only 0.1% of the neurons, one can still recover χ=2. However, this approach fails to simultaneously restore all exponents while maintaining the crackling-noise relationship (see Fig. 2A and Suppl. Fig. S1) because the point of concurrent rescue lies within the unobservable regions of parameter space. Decreasing sampling further to f=0.01% (100 neurons out of 1 million neurons), prevents rescue of χ=2.

Temporal coarse-graining does not rescue the ‘crackling noise’ relationship of $\chi_{CN}$ (Equation 2). The DCC, introduced recently as the difference between the data-based value of χ (Equation 3) and $\chi_{CN}$ should measure close to 0 if both estimates agree. As can be seen in Figure 4, co-regions with DCC=0 and χ=2 exist in the fully-sampled system (Fig. 4; asterisk). In contrast, no such co-alignment is found for subsampled systems and temporal coarse graining fails to establish a DCC=0 regime for most values of k and θ (Fig. 4; square, triangles) and accordingly DCC=0 does not reflect a proper rescue of the true χ=2 under these conditions.

### Systematic rescue of the scaling exponent for parabolic avalanches in frontal cortex of awake mice

**Figure 5.**
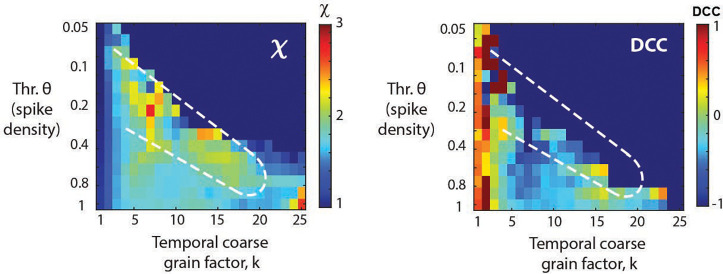
Parabolic avalanches in ongoing activity of frontal cortex exhibit threshold and temporal coarse-graining rescue of scaling exponent χ=2 in line with critical model dynamics. Scaling exponent, χ, and crackling noise deviation, DCC, as a function of the spike density thresholds and temporal coarse grain factor, k(n=5 mice; 2-photon imaging of ongoing activity in frontal cortex (anterior cingulate and medial prefrontal cortex). At low k, an underestimation of χ correspondents to noisy, low values of DCC. At higher k, and threshold θ, rescue of χ to 2 corresponds to high values of the DCC. Note the relationship between k and θ at which χ≅2 is similar to that identified in the critically balanced, E-I model (*broken line*).

We demonstrate this performance of χ and failure of the DCC in ongoing cellular 2-photon imaging of awake mice from the anterior cingulate cortex (for details see also [9]). In Figure 5, we demonstrate the robust rescue of the scaling exponent χ=2 in our data set for different spike density threshold levels (Fig. 5; for details see Methods and [9]). We find that the recovery of χ=2 exhibits a θ vs. k dependency as predicted by our simulations, whereas the DCC is variable and noisy (Fig. 5). Similar results were obtained when using a z-scored threshold (Suppl. Fig. 2).

## DISCUSSION

The exploration of criticality in neuronal systems, particularly focusing on neuronal avalanches, has been subject to extensive investigation and debate (for review see [18, 51, 57–60]). This study addresses the intricacies and challenges associated with estimating scaling relationships in the brain, especially in the presence of fractional subsampling. Our key finding of significance revolves around the retrieval of scaling exponents under fractional subsampling conditions. Our findings illustrate that temporal coarse-graining facilitates the precise estimation of the critical scaling exponent (χ≈2), even when observing a minimal fraction (0.1%) of the network’s neurons. This discovery carries substantial implications, indicating the potential to accurately estimate critical scaling characteristics despite severe subsampling constraints. Previous work on similar directed percolation (DP) models demonstrated a degradation of the scaling exponent to 1.3 with spatial subsampling of 10% and below [38]. Subsampling has also been shown to mimic apparent population independence of correlations, affecting criticality markers such as specific heat [61]. We note that in our model, spatial correlations’ dependencies cannot be explored due to the all-to-all connectivity.

### Temporal coarse-graining when avalanche periods are unknown

Temporal coarse-graining has been proposed previously as a potential solution to rescue the size exponents of avalanches in subsampled systems [40] assuming an infinite separation of time scales. This assumption would provide the experimentalist with the information to separate sequentially occurring avalanches, which is unrealistic in the study of natural systems. In contrast, our present analysis demonstrates that temporal coarse-graining can rescue the scaling exponent in models without a clear separation of time scales for fractional sampling as low as 0.1%, suggesting its potential value in studying highly subsampled experimental data falling within the DP class. Our study introduces threshold above the Poisson noise floor to approximate separation of successive avalanches and uncovers a trade-off between subsampling and thresholding. Elevating the threshold in a fully sampled network is akin to subsampling the network and adopting a lower threshold. This highlights the interplay between these two factors and their cumulative influence on criticality assessments. By exploiting this trade-off, we apply our methodology to cellular-resolution 2-photon imaging data obtained from the anterior cingulate cortex of awake of mice. We underscore the consistent recovery of the scaling exponent to its critical value of 2. Recently, avalanche detection methods have been introduced that replace temporal discretization with a continuous time model, though it is currently not clear how these model compare with experimental data and subsampling conditions [62].

We note that temporal coarse graining differs from a renormalization procedure, which for neuronal avalanches has been shown to preserve estimates of maximal mutual information as one coarse-samples the spatial scale under fairly complex macroscopic sampling conditions of the LFP [63]. The temporal coarse graining applied here rather addresses a stochastic subsampling bias as detailed previously [9].

### Limits in rescuing the scaling exponent under subsampling conditions

Our simulations also clearly show that subsampling in discrete models renders certain parts of the phase space inaccessible (*c.f.* Fig. 4), preventing the recovery of the scaling exponent under highly subsampled conditions (*f* << 0.1%). A similar limitation has been demonstrated for subsampling in the temporal domain, particularly when the temporal precision required exceeds the temporal resolution of the model [38]. These spatial and temporal subsampling constraints are particularly relevant to experimental data, which often faces limitations in both temporal and spatial resolution. Similar constraints have been found for heavy subsampling of network topologies, particularly when estimating heavy-tail or scale-free distributions of graph properties [64–66]. Insights into under-sampled critical branching processes have been used to understand avalanche statistics obtained from extracellular spike recordings using microelectrode arrays in the anesthetized rat [67]. Despite these limits imposed by extreme subsampling, our finding holds promise for experimental neuroscientists contending with the practical constraint of recording from only a subset of neurons within neural circuits. It suggests that, with careful consideration of experimental parameters, criticality assessments can remain feasible and informative, offering valuable insights into the fundamental mechanisms governing neural synchronization.

### The DCC and crackling noise relationship fails to recover the scaling exponent under subsampling conditions

The crackling noise relationship [49–51] has been increasingly proposed and used to quantify the distance of the observed system from its critical regime (e.g. labeled as the Deviation from Criticality Coefficient (DCC) (e. g. [39, 42]) . However, as outlined by [36] with respect to the general claim by [39], random processes can exhibit a DCC close to 0 thus limiting general claims of this approach to identify critical dynamics even in fully sampled systems. In the present study, we clearly show that the DCC approach leads to biased estimates under subsampling conditions. We also emphasize the high sensitivity of the DCC metric to temporal coarse-graining, which appears noisy in both our subsampled model and experimental data. Additionally, the DCC metric exhibits a singularity as the denominator of $\chi_{cn}$ passes through 1, complicating its use as an accurate marker of critical systems. A major confounding factor when using the DCC approach is the assumption that slope estimates from size and duration distributions equate with critical exponents. This is obviously not the case, as various analysis parameters such as the temporal resolution Δt and spatial grid sampling distance Δd relate to the obtained power law slopes in a predictive manner [7, 19], which has to be taken into account when estimating critical exponents from experimental measures.

Our analysis further highlights the scaling exponent’s sensitivity to synchronization thresholds. As the threshold increases, we observe a noticeable deviation of the scaling exponent and the crackling noise ratio $\left.{(\chi }_{cn}\right)$ from the expected value of 2 (see also [13]). This sensitivity underscores the importance of careful consideration of thresholding strategies in experimental setups. Thresholding of time-series can lead to a change in the avalanche size exponent itself as demonstrated in simulations of critical dynamics [13]. In general, a higher threshold can slightly increase α, however, the effect on estimating χ appears to be small. In fact, Capek et al. [9] show in their experimental analysis that including or excluding the subthreshold regime for size estimates provides narrow upper and lower boundary estimates of χ that center around 2 (see their supplemental figure 6 in [9]). We suggest that the selection of thresholding parameters should ideally be guided by a judicious equilibrium that ensures the preservation of genuine synchronization events while minimizing the introduction of noise, especially when the accurate estimation of scaling exponents is the objective.

### Synchronization, coincident firing and delayed neuronal correlations

Spatiotemporal synchronization in neural systems traditionally focused on phase-locked oscillation. However, our system stands out due to the absence of a dominant frequency, presenting a unique scenario. This peculiarity gains significance when considering ‘noise correlations,’ which encapsulate the intrinsic non-stimulus-induced correlation structure among neurons. These correlations exert a notable influence on the decoding capabilities of neuronal populations, constituting a pivotal aspect of neural information processing [68, 69].

In this study, we scrutinize scale-free neuronal avalanches as the context for exploring neuronal synchrony and criticality. Neuronal avalanches furnish a comprehensive framework for comprehending neuronal synchronization, spanning from local clusters of neurons to the global scale of the entire brain. As demonstrated, the quantitative analysis of neuronal avalanches crucially hinges on the choice of thresholds and temporal coarse-graining. This dependence is similarly observed in the analysis of spatial and temporal neuronal correlations, which are substantially influenced by spatial and temporal coarse-graining, respectively [70, 71]. Nevertheless, as depicted in Figure 3, the restoration of the scaling exponent, χ, does not straightforwardly relate to an increase in pairwise correlations. This underscores the restoration of χ to its critical value of 2 as an indicator for critical systems. Our findings bear implications for neuroscience and complex systems. Neuronal avalanches serve as valuable indicators of neural synchronization within the cortex. The precise estimation of scaling exponents, even under subsampling conditions, is imperative for elucidating the mechanisms governing neural synchronization in both physiological and pathological states. It bolsters the proposition that the brain operates near critical points, aligning with the broader concept of self-organized criticality observed in various complex systems.

### Neuronal cell assemblies in the form of parabolic avalanches

In conclusion, our research aims to contribute significantly to the ongoing discourse surrounding criticality in neural systems. We address the challenges and potential solutions associated with estimating scaling relationships in neuronal avalanches, especially when faced with subsampling and thresholding constraints. We present our results as a framework for future investigations into the nature of criticality within neural networks, paving the way for deeper insights into the fundamental principles governing both normal brain function and dysfunction.

## Methods

### Model Topology

In this work, we employed the model originally proposed by Girardi-Schappo et al. [46] and subsequently modified in Capek & Ribeiro et al. [9]. This model represents a critically balanced system of integrate-and-fire (IF) neurons, where the excitatory and inhibitory neurons maintain an equilibrium. Our neuronal network consisted of N = 10^6^ stochastic, non-leaky IF neurons, with a fully connected architecture. To reflect the prevalence of excitatory neurons in the cortex, we set the ratio of excitatory to inhibitory neurons to 4:1. The connectivity matrix, denoted as W, was initialized in a manner such that all outgoing synapses from excitatory neurons had equal strength, with $W_{EE}=W_{IE}=J$. Similarly, the inhibitory neurons had equal strength for their outgoing synapses, with $W_{\text{II}}=W_{EI}=-gJ$. Here, the balance parameter, g, plays a crucial role in tuning the model towards an E/I-balanced state exhibiting critical dynamics. For our network, the critical value of $g,\;g_{c}$, was determined to be 3.5. When g exceeds $g_{c}$, the network shifts towards an inhibition-dominated regime with subcritical dynamics, whereas values of g below $g_{c}$ cause the network to exhibit an excitation-dominated regime with supercritical dynamics.

### Model Dynamics

Each neuron in the model was described by two variables. The Boolean variable, X, indicated whether a neuron was firing or in a quiescent state at time t. Specifically, X(t)=1 represented firing, while X(t)=0 denoted quiescence. The membrane potential, V, influenced the firing probability and evolved according to the following equation:

$$V_{i}(t+1)=\left(\mu V_{i}(t)+\sum_{j=1}^{N}W_{ij}X_{j}(t)\right)\left(1-X_{i}(t)\right)$$

In the equation, μ represented the leakage parameter, which we set to 0 (non-leaky) for our simulations. The term $\left(1-X_{i}(t)\right)$ introduced an absolute refractory period of Δt, allowing the voltage to reset after a spike occurred (when $X_{i}(t)=1$). Additionally, the probability of a neuron firing linearly increased with V and was given by:

$$P(X=1|V)=\left\{\begin{array}{l} V,\Gamma V<1\\ 1,\Gamma V\geq 1 \end{array}\right.$$

Here, Γ represented the neuronal gain, which we fixed at 1 to support probabilistic behavior. Notably, this network exhibited significant non-conservativeness, with energy dissipation occurring through inhibition and spike collision.

Additionally, we note that this model includes an absorbing quiescent state, and therefore, the network activity is initiated by very low-rate independent Poisson processes applied to each neuron. Specifically, the Poisson process triggers approximately ~20 neurons (0.002%) on average per time step, which is negligible compared to the system size of 10^6^ neurons.

### Avalanche Statistics

To comprehensively investigate the resulting population activity, we run 100 simulations of the network over an extended duration of 10^6^ time steps for a total of 10^8^ time steps. For the case of full sampling, we compute the sum of activity across the entire population of N = 10^6^ neurons. However, to explore various sampling scenarios, we consider a random subset of N*f neurons, where f represents the sampling fraction. Following this, a thresholding procedure is applied to the time-series data, setting all neuronal activity below the threshold value θ to 0. We have the option to analyze the data within the thresholded time-series in their original form or subject them to temporal coarse graining. When employing coarse graining, each generation represents the sum of activity over k consecutive time bins, with k representing the coarse graining factor. This approach enables us to investigate network dynamics across different temporal scales and analyze resulting patterns. Consequently, we collect the sizes and durations of contiguous epochs from the time-series and examine their distributions.

### Power-law fitting

In the context of an ideal, infinitely sized critical system, the distribution of size, duration, and average size as a function of duration adheres to perfect power laws. However, it is important to consider statistical anomalies, finite size effects, and the influence of the low Poisson drive in our simulations when analyzing these distributions. Therefore, a cautious approach is required for their analysis within the model. In the average size versus duration analyses, we observed that the distributions could be reasonably approximated by a double power law. For this purpose, we employ the fitting function introduced by Capek & Ribeiro et al. [9]:

$$S(d)=\frac{Cd^{\sigma_{1}}}{{\left(1+(d/\Phi {)}^{\gamma }\right)}^{\left(\sigma_{1}+\sigma_{2}\right)/\gamma }}$$

This function exhibits an initial slope $\sigma_{1}$ that transitions to a second slope $\sigma_{2}$ around the point d=Φ. The parameter γ governs the abruptness of this transition and has been set to 4 for all the analysis presented. The remaining parameters are allowed to adjust to the data, and all fits were performed in log-space. In all plots presented in this paper, $\sigma_{1}$ is employed as the representative slope, while $\sigma_{2}$, which is typically associated with finite-size effects, is not discussed in this context.

We use the cutoff, Φ that we obtain from the scaling curves to analyze the individual size and duration distributions. The size exponent, β, is the slope of the size distribution in log-scale with a lower cutoff which is safely set to be greater than the Poisson noise level (>100) and an upper cutoff equal to the average size corresponding to the elbow point, Φ. For the duration exponent, β, we do a similar analysis, with a lower duration cutoff of 3 time-bins and an upper cutoff equal to Φ.

### Correlation analysis

In our examination of the mean pairwise correlation within the system, we focus on a smaller temporal segment of the simulated network (consisting of 10,000 time steps) to facilitate analysis of large neuron raster datasets. For each neuron pair (designated as an ordered pair [i,j]) within the network, we compute the time-lagged correlation between the activity of neuron i and that of neuron j, shifted by one time step, k*Δt. The mean correlation across the system is then determined as the average correlation coefficient computed for all such neuron pairs. This correlation analysis spans temporal bins ranging from 0 to 100 and spatial sampling values from 0.01% up to 1%. However, due to the quadratic increase in duration and memory requirements with system size, higher sampling rates become impractical for storing raster data and conducting pairwise analyses.

### Ongoing activity in contralateral ACC/mPFC monitored with jRGECO1a.

Mice (C57BL/6; Jackson Laboratories; age >6 weeks) were injected with a viral construct to express jRGECO1a in cortical neurons using Syn promotor. Chronic 2PI started after ~2 weeks in the contralateral ACC/mPFC at an estimated depth of ~150–300 μm using a microprism. Recordings were collected over the course of several days from n = 5 mice (3 males, 2 females; age 8–20 weeks) with jRGECO1a expression (n = 17 recordings; 30min each). Recordings were conducted over the course of several weeks and analyzed separately for each mouse. 2PI images were acquired by a scanning microscope (Bergamo II series, B248, Thorlabs Inc.) coupled to a pulsed femtosecond Ti:Sapphire 2-photon laser (Chameleon Discovery NX, Coherent Inc.). The wavelength was tuned to 1120 nm to excite jRGECO1a. The field of view was ~450 um × 450 μm. Imaging frames of 512 × 512 pixels were acquired at 45.527Hz by bidirectional scanning of a 12 kHz Galvo-resonant scanner. The average power for imaging was <70mW, measured at the sample. Obtained tif-movies in uint16 format were rigid motion corrected via the python-based software package suite2p [72]. Registered images were denoised using machine-learning-based, DeepInterpolationLecoq [73]and then semi-automatically processed by suite2p for ROI selection and fluorescence signal extraction. For each labeled neuron, raw soma and neuropil fluorescence signals over time were extracted for each ROI. Spiking probabilities were obtained from neuropil corrected fluorescence traces (F_corrected = F_ROI – 0.7*F_neuropil) via MLspike (https://github.com/MLspike) by utilizing its autocalibration feature to obtain unitary spike event amplitude, decay time, and channel noise for individual ROIs.

## Supplementary Material

Supplement 1
