# Supplementary material for: The recovery of parabolic avalanches in spatially subsampled neuronal networks at criticality
Source: bioRxiv. 2024 Feb 28:2024.02.26.582056. Preprint. [Version 1] doi: 10.1101/2024.02.26.582056 (PMC10925085; doi:10.1101/2024.02.26.582056)
Supplement: Supplementary file 1 [file NIHPP2024.02.26.582056v1-supplement-1.pdf]

bioRxiv preprint doi: https://doi.org/10.1101/2024.02.26.582056; this version posted February 28, 2024. The copyright holder for this preprint (which was not certified by peer review) is the author/funder. This article is a US Government work. It is not subject to copyright under 17 USC 105 and is also made available for use under a CC0 license.

SUPPLEMENTARY INFORMATION

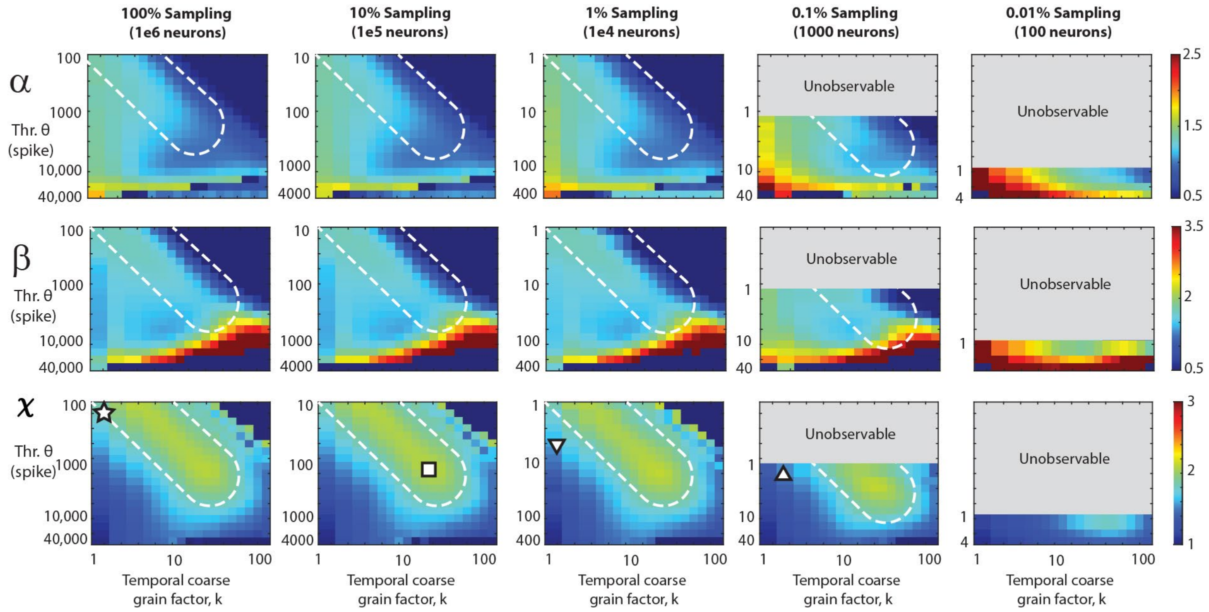

Suppl. Fig. S1. Consolidated view of the exponents α, β and χ as a function of threshold and temporal coarse-graining for different sampling fractions.

(**A**) Consolidated view of α, β and χ as a function of θ and k for different values of f. For f = 100%, $\chi \cong 2$ for low θ and k (*star*) but as we make the data sparser by increasing the threshold (or reducing the sampling), we need a higher coarse graining factor, k, to compensate and rescue χ back to 2 (*square*). Bottom row is replotted for χ from Figure 4 for ease of comparison with the corresponding size slopes, α, and duration slopes, β. *White dotted region*: visual guide for χ close to 2 for increasing θ and k. The grey parts of the plots for f = 0.1% and 0.01% respectively are unobservable parameter regions since they would require fractional thresholds, below the 1 spike minimum resolution of the model (*cf.* Figure 4).

bioRxiv preprint doi: https://doi.org/10.1101/2024.02.26.582056; this version posted February 28, 2024. The copyright holder for this preprint (which was not certified by peer review) is the author/funder. This article is a US Government work. It is not subject to copyright under 17 USC 105 and is also made available for use under a CC0 license.

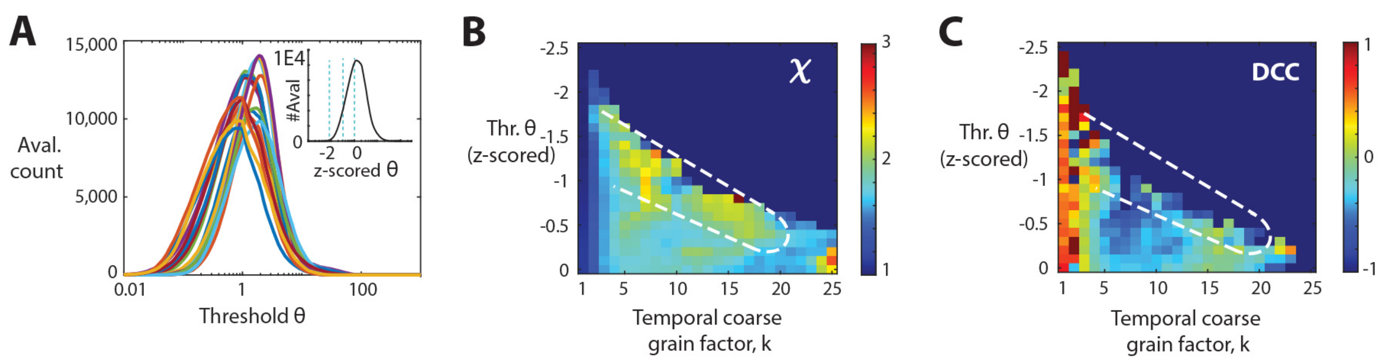

Suppl. Fig. S2. Parabolic avalanches in ongoing activity of frontal cortex exhibit threshold and temporal coarse-graining rescue of scaling exponent χ = 2 in line with critical model dynamics: z-scored threshold.
(**A**) Number of avalanches as a function of threshold for 17 different recordings of ongoing neuronal activity in anterior cingulate frontal cortex. Inset highlights the z-scored threshold which is calculated independently for each recording. Data pooled from 5 mice (3 males, 2 females) with jRGECO1a expression for a total of 17 recordings of 30 mins each (2-photon imaging).
(**B, C**) Scaling exponent and DCC as a function of the z-scored thresholds instead of absolute spike density threshold (*cf*. Figure 5) and temporal coarse grain factor, *k*.
